# “Veterinary medicine is not finished when I have diagnosed an incurable disease, that’s when it starts for me.” A qualitative interview study with small animal veterinarians on hospice and palliative care

**DOI:** 10.3389/fvets.2024.1440404

**Published:** 2024-09-05

**Authors:** Svenja Springer, Shannon Axiak Flammer, Christian Dürnberger

**Affiliations:** ^1^Department of Interdisciplinary Life Sciences, Messerli Research Institute, University of Veterinary Medicine Vienna, Vienna, Austria; ^2^Department of Clinical Veterinary Medicine, Vetsuisse Faculty, University of Bern, Bern, Switzerland

**Keywords:** small animals, dogs, cats, end-of-life care, palliative medicine, interview study, veterinary medical ethics

## Abstract

In the wake of recent medical developments in small animal practice, curing animals of their illnesses and restoring their health can be realized better than ever before. However, the growing medical possibilities are also leading to an increase in demand for better care for patients suffering from terminal illnesses. Consequently, the field of animal hospice and palliative care has become increasingly available, enabling veterinarians to optimize the quality of life of patients, such as dogs and cats, who no longer have a prospect of full recovery. Using qualitative, semi-structured interviews with 20 small animal veterinarians involved in hospice and palliative care, we investigated the factors that motivate veterinarians to become involved in hospice and palliative care and explored the importance of relationships, communication, time and infrastructure in this area. Findings show that personal experiences with their own pets or during training or work life motivated veterinarians to provide this service. Although veterinarians highlighted the importance of empathetic-driven relationships, they were aware that keeping an emotional distance from the patient and caregiver is significant to provide successful care. Further, veterinarians emphasized their high investment of time that resulted primarily from the increased frequency and provided opportunities to communicate with caregivers. The overall conclusion is that having time for patients and the patients’ caregivers is one of the most important aspects of work in this field. However, as it will be also shown, veterinarians must consider aspects of self-care management by reflecting on their own time and energy resources while caring for animals and their caregivers.

## 1 Introduction

Animal caregivers, seeking veterinary care for their dog or cat, benefit from a wide range of diagnostic and therapeutic options that are similar to those in human medicine (1–4). Advanced imaging, for example, allow diseases to be detected in early stages (5). The implementation and use of chemo- and radiotherapy make it possible to cure cancer patients who could not be treated just a few decades ago (6). In the wake of these recent medical developments, curing animals of their illnesses and restoring their health can be realized better than ever before. At the same time, however, the growing medical possibilities are also leading to an increase in demand for better care for patients suffering from terminal illnesses who have no prospect of a cure (4). As a result, the field of animal hospice and palliative care has come to the fore (7, 8). In contrast to aim for curing the animal, the focus of hospice and palliative care lies in the management of pain and clinical symptoms to achieve the best possible quality of life of a patient, who no longer has any prospect of recovery (4, 9). Shearer (7) provides clear definitions for both hospice and palliative care, which highlight not only important goals but also differences between them. Whereas “[p]alliative care addresse[s] the treatment of pain and other clinical signs to achieve the best quality of life regardless of disease outcome” (7), “[h]ospice is a specialized form of a palliative care that focuses on caring for patients in the end stages of an illness, near death [and] relies on a philosophy of care that embraces death as a normal process” (7).

These definitions highlight specific goals and requirements of animal hospice and palliative medicine that are not fully fulfilled by “traditional” fields of small animal medicine. Herewith, we consider, e.g., general medicine, surgery or internal medicine as traditional fields which have become firmly established in small animal practice. Even though “veterinarians have historically provided many of the defining elements of hospice and palliative care” (4), the institutionalization of hospice and palliative care seems to be increasingly relevant by focusing on patients’ well-being and quality of life with their diseases (9). Against the background of the specific goals and requirements related to the field, it has been hypothesized that veterinarians experience a shift from primarily “curing” to primarily “caring” professionals in this context (9). In addition, this shift can be further explained by the fact that veterinarians provide not only medical and supportive care for the animal, but further offer emotional support to the caregiver (10–12). Several scientific publications explicitly argue for further consideration of these emerging shifts (4, 7, 10–13), however, to the best of the authors’ knowledge, most of these discussions are mainly based on theoretical assumptions and anecdotal knowledge. While there are only a few empirical studies among veterinary professionals, these focus on specific aspects such as euthanasia including client grief (14), general challenges in end-of-life care (13, 15) or a comparison between professionals from human and veterinary medicine in end-of-life decisions (13). Thus, there are currently no systematically conducted studies that provide empirical insights into a veterinarian’s motivation to build competencies in the field of hospice and palliative care and possible changes experienced in this field compared to traditional fields. Against this background, we aim to fill this research gap by focusing on an empirical investigation of four aspects that are assumed to be of particular importance for hospice and palliative care (9).

The first aspect, “relationships,” focuses on the specific trialogue between the veterinarian, caregiver, and patient (16, 17), which has an impact on decision-making-processes in veterinary medicine. Especially in the field of hospice and palliative care, relationship-based care is an important requirement (7, 11), as end-of-life decisions are often shaped by emotional relationships. Due to the actual or expected grief of the caregiver and the increase in visits to the veterinarian, it is expected that the relationships between caregivers and the animal as well as between caregivers and veterinarians intensify when caring for patients at the end of life, and that a close emotional bond between the caregiver and the animal is a prerequisite for the successful care of patients in this area.

The second aspect, “time,” deals not only with the time needed for care, but also with how the remaining time at the end of an animal’s life is perceived by those involved. In comparison to general veterinary practice, where in the case of a curable disease the restoration of health usually determines the end of veterinary care, the time frame in animal hospice and palliative care is determined by other reference points: It begins with the incurable diagnosis and ends with the death of the animal. In addition to the duration of treatment, the frequency and time required for hospice and palliative care must also be considered (9). Further, not only the quantity of time, but also the quality of the remaining time comes into focus in this working field since the time span determined by the terminal diagnosis and the death of the animal (whether euthanasia or natural death) influences the perceived quality of the remaining time. Consequently, veterinarians must adapt their business concept to the increased time required for hospice and palliative consultations, so that they can effectively integrate them into their professional lives and reconcile it with their private lives.

Even though veterinarians repeatedly describe their work as essentially communicative (18), recent studies on communicational aspects focus primarily on a medical context in which the aim is to provide “traditional” curative treatment for an animal (19–22). Given that the practice of animal hospice and palliative care is often associated with emotional challenges, good communication strategies form an important basis for successful care (12). In this respect, “communication” is considered as the third important aspect, in which changes to the frequency, topics and channels of communication are expected when compared with general practice.

The fourth aspect, “infrastructure,” considers different working environments of veterinarians, interdisciplinary collaboration and training opportunities that are important for the successful institutionalization of the field in small animal practice. Important infrastructural aspects such as the type of practice, the interdisciplinarity of the teams (e.g., including social workers) and the type of training institutions (e.g., university or association-based) must be reconsidered, as has also happened in palliative and hospice care in human medicine in recent decades (23, 24). In addition to the different working environments (practice versus working in private households), this also includes interdisciplinary cooperation (colleagues, psychologists, social workers, etc.).

While making use of these above-mentioned four aspects to guide our empirical investigation, this study followed a clear thematic orientation. Semi-structured individual focused interviews (25) with small animal veterinarians working in the field of hospice and palliative care were conducted in Austria, Germany, and Switzerland. In contrast to open narrative interviews, focused interviews are based on a comprehensive concept and serve not only to generate hypotheses, but also to verify them (25). In addition, we decided to include these three countries in the interview study because they are all German-speaking, have virtually identical veterinary educational programs, and show parallels in the structure of their veterinary professions (e.g., a high percentage of self-employed veterinarians, together with a low percentage of corporate-owned practices).

The main aim of this qualitative interview study was to provide a detailed mapping of how small animal veterinarians experience their work in hospice and palliative care. This was achieved based on answers to the following research questions: (1) What motivated veterinarians to provide and/or specialize in animal hospice and palliative care? (2) How do veterinarians experience relationships between the animal caregiver and the animal as well as the caregiver and themselves in the context of hospice and palliative care? (3) What role does time play when caring for a chronically ill patient and/or a patient near to death? (4) How and about what do veterinarians communicate with their clients in hospice and palliative care? (5) And, how do veterinarians describe their working environment and what infrastructural requirements are necessary to successfully offer animal hospice and palliative care?

## 2 Materials and methods

This paper is based on individual focused interviews (25) of 20 small animal veterinarians, resident in Germany (*n* = 8), Switzerland (*n* = 7), and Austria (*n* = 5), who specialized in the field of hospice and palliative care [certificated by the International Association for Animal Hospice and Palliative Care (IAAHPC)] and/or explicitly mentioned and advertised it on their practice website.

### 2.1 Overview of small animal veterinarians with a focus on hospice and/or palliative care and the selection of study participants

A comprehensive search of online classified directories was conducted for Germany,^1^ Switzerland,^2^ and Austria^3^ in June and July 2022. The search of online classified directories aimed to gain an overview of veterinarians as well as practices that explicitly advertised and mentioned the provision of “palliative care,” “palliative medicine,” and/or “hospice service” on their website. Since this study focuses on this specific working field, we did not include websites that mentioned the service of “euthanasia” only. Even though euthanasia plays an important part when it comes to end-of-life decisions, the aim of the current study was to investigate particular changes, chances and challenges veterinarians face in the provision of hospice and palliative care.

In total, 3,007 websites were reviewed. Websites that were duplicated or that displayed large animal practices or other business websites were removed from the list, resulting in a total of 2,204 websites (Table 1). Results of the online search showed that a total of 52 websites (2.4%) mentioned the service of hospice and/or palliative care.

**Table 1:** Results of an online search for websites of veterinarians, practices and/or clinics in the small animal sector that explicitly mentioned the provision of palliative care and/or hospice service on their websites in Germany, Switzerland, and Austria.

Country	Reviewed websites	Included websites	Advertised palliative care and/or hospice service on the website
Germany	2,370	1,682	36 (2.14%)
Switzerland	348	233	8 (3.4%)
Austria	289	289	8 (2.7%)
Total	3,007	2,204	52 (2.4%)

Even though online classified directories provide a good overview of practicing veterinarians and practices, it is important to note that a coverage error exists, since not all veterinarians may have a practice website or are not displayed in the used directories. To minimize the coverage error, an additional online search via google.at was conducted in August 2022. Under the use of relevant search terms, including “veterinarian,” “palliative,” “hospice,” and “small animal practice,” a total of 37 additional veterinarians, practices and/or clinics in the small animal sector (Germany: *n* = 28; Switzerland: *n* = 2; Austria: *n* = 7) were identified that were not displayed in the used classified directories.

Veterinarians were only recruited if they practiced small animal medicine and stated palliative care and/or hospice service on their website. The interview study was planned to further include professionals with different fields of interests and specialization (e.g., certification in homeopathy, specialization in hospice and palliative care by the IAAHPC) and format of practice (e.g., mobile practice), as we hypothesized that these aspects may affect their work. Thus, the sampling strategy involved participants from several strata: (a) form of practice (practice versus mobile practice); (b) type of specialization and post-graduate education; (c) years of work experience; (d) level of urbanization (from rural region up to cities with more than 1 million inhabitants) and (e) working place (country and federal state).

### 2.2 Recruitment process and study participants

At the beginning of December 2022, invitations were emailed to 20 selected veterinarians (Germany: *n* = 9; Switzerland: *n* = 5; Austria: *n* = 6) based on the above-mentioned criteria. All the veterinarians were personally contacted by telephone one to two weeks later (mid-December 2022). Various aspects in relation to the recruitment of participants, e.g., diversity in form of practice, type of specialization, years of work experience, degree of urbanization and working place, were considered to obtain a broad spectrum of the experiences and thereby increase the chance of transferability (26). To this effect, we applied a purposive sampling strategy, namely quota sampling (27), with the aim to get at least 3–4 participants from each sub-group of the variables listed in Table 2. The sample size target was 20 participants, as we expected to reach data saturation in discussed aspects that are of relevance in the field of hospice and palliative care. However, in the recruitment process—that was based on the results of our online search—it was difficult to find male and employed veterinarians as well as veterinarians who worked in mobile practice only. It turned out to be very difficult to further recruit suitable candidates that fall into these categories. We therefore decided to accept the lower number of participants in these sampling strata.

**Table 2:** Descriptive characteristics of interview participants at the time the interviews took place.

Descriptive characteristics		Number of participants (*N* = 20)
Country of work	Germany	8
	Switzerland	7
	Austria	5
Residential area	City of over a million citizens (<1 Mio citizens)	2
	Large city (>100.000 citizens)	3
	Medium-sized town (20.000–100.000 citizens)	6
	Small town (5.000–20.000 citizens)	2
	Rural town (<5.000 citizens)	7
Work experience (in years)	5–15	4
	16–25	8
	26–35	8
Gender	Male	2
	Female	18
Specialization and post-graduate education	No specialization and post-graduate education	9
	Certificated or in training in palliative and hospice service	3
	Education and/or certification in homeopathy	5
	Other post-graduate education (e.g., internal medicine)	3
Practice type and employment status	Self-employed mobile veterinarian	3
	Self-employed veterinarian	16
	Employed veterinarian	1

After this first phase of the recruitment process, 16 veterinarians agreed to participate in the interview study. Suitable replacement candidates were selected for veterinarians who declined the invitation, did not respond, or could not be reached by telephone within three weeks of the e-mails being sent. Consequently, during the second recruitment phase, four further veterinarians were contacted (Germany: *n* = 3; Austria: *n* = 1), and one further veterinarian agreed to participate. In mid-March 2023, two further veterinarians working in Switzerland were invited to contribute to the study. Both accepted one week after the invitation. In total, 26 veterinarians were contacted during the recruitment process, and 19 veterinarians confirmed their participation. In January 2023, two pilot interviews were conducted with one veterinarian working in Austria and one veterinarian working in Switzerland (see sub-section data collection procedure and structure of the interviews). Due to the smooth running of one of the two pilot interviews, it was decided to include this interview in the final sample of the study.

Participants were offered 50 € compensation for participating in the interview study. Following the ethical approval process at the University of Veterinary Medicine, Vienna, the study was submitted for ethical approval to the Ethics Committee of the Medical University of Vienna before the start of the recruitment process. After reviewing the study, the requirement for further ethical approval was waived by the committee. Before the study, all participants were provided with information about the type of data that would be recorded in the study and how it would be handled, that participation was voluntary and that they were free to withdraw their consent at any time during or after the study. All participants provided written consent and no participant took the option of withdrawing their consent during or after the study.

### 2.3 Data collection procedure and structure of the interviews

All 20 interviews were conducted in German and online, by using a web conference tool (Cisco Webex) through which they were videorecorded. As one interviewee had technical issues with the tool, the interview was conducted and recorded via mobile phone without video recording. The duration of interviews ranged from 48 to 100 min, with an average duration of 73 min. All interviews followed a semi-structured interview guide. This consisted of four parts including six themes (Supplementary material 1). Depending on the interviewee, the formulation of individual questions was slightly changed and adapted to suit the participant and his or her working background. However, the process of each interview was the same in terms of structure and overall content. A detailed description of parts and themes of the interview guide that are relevant for the present study are presented in Supplementary material 2.

The steps and contents of the interview guide were piloted by two veterinarians. One Austrian veterinarian explicitly mentioned the service of palliative care on the website, and one Swiss veterinarian who was specialized in the field of hospice and palliative care and further, worked as a mobile veterinarian. Two authors of the present paper conducted one pilot interview each (SS, CD). Besides the incorporation of a few comments and suggestions into the final version of the interview guide, the authors discussed experiences and challenges that occurred during the pilot interviews. In total, one author (SS) conducted 18 interviews and a second author (CD) conducted two interviews.

### 2.4 Data analysis

Recordings were transcribed verbatim and coded using the MAXQDA 2020 (version 20.4.2) software program (Berlin, Germany). Following the template organizing style (28), categories and codes were created and formulated based on the six themes and key aspects of the interview guide including motivation, relationships, communication, time, infrastructural requirements and dying and death of animals (Supplementary material 1), the research questions, and the hypotheses of the project. A first and second cycle of coding were conducted for the analysis of the data gained from the 20 interviews (29). Using a deductive approach, the overall aim of the first cycle of coding was to summarize segments of data and categorize similar data units (29). The initial code list for the first cycle of coding contained nine categories with a total of 40 codes. During the first coding cycle of two interviews (conducted by SS and CD), this initial list was adjusted: two new codes were added, and other codes were adjusted to make them more applicable for the analyses. These alterations chiefly followed an inductive approach based on gained data. Hence, the final version of the code list included nine categories with 42 codes (Supplementary material 3).

The coder (SS) mainly used descriptive coding, hypothesis coding, and holistic coding to organize and summarize the text segments (29). The coding was mainly done with the transcribed text. The coder consulted the recordings in the event of uncertainties (e.g., positive or negative meanings of statements). Categories and in particular codes were continuously discussed by the project team (SS and CD) to ensure relevance and use of codes, especially during the first cycle of coding. In a second cycle of coding, the initial results were grouped into smaller categories and clusters to gain more meaningful units for subsequent content analysis (29). The present interview study was a qualitative social science study. The aspiration was not to make statistical generalizations about a background population, but rather, to obtain in-depth accounts of beliefs, attitudes, everyday practices, and decisions that underly decision making in the context of hospice and palliative care.

## 3 Results

Results of this interview study reveal aspects that enhance our understanding of the source of veterinarians’ motivation to build competences in the field of animal hospice and palliative care, and the extent to which relationships, communication, time and infrastructure play a role in the care of chronically and/or terminally ill patients.

### 3.1 Source of motivation to provide and/or be specialized in the field of animal hospice and palliative care

#### 3.1.1 Experiences with own animals

One aim was to identify the source of motivation for providing and/or specializing in the field of animal hospice and palliative care. Several participants mentioned their previous experiences as pet owners. For instance, one veterinarian stated: *“We all learned – me as well – quite clearly at university that when euthanizing to not make a fuss, let go, and make reasonable decisions. And then I realized how much I suffered from the euthanasia of my own animals. We spoke to people who felt the same way. And that was one of the reasons to simply start thinking outside the box.”* (Interview 4, Position (Pos.) 45). Further, participants of this study highlighted their negative experiences with the veterinarians who provided medical care for their animals. As one specialized interview partner reported: *“Well, we had a dog that got relatively old, almost 15 years old, and was a large Labrador mix and had pretty much all the diseases of old age, […] so we realized how little information there actually is as pet owners […]. [S]o there were still a lot of vets who did not know that much about diseases of old age, who just could not help you that much. […] And that’s exactly why I feel such a need to improve that, because it’s such a shame when 15 years that were nice end up being so traumatic […].”* (Interview 2, Pos. 31).

#### 3.1.2 Experiences during veterinarians’ training programs

Another source of motivation for the provision of hospice and palliative care lies in the occasional negative experiences of veterinarians during training. Interviewees criticized the observed handling of chronically ill and/or old patients with a poor prognosis and emphasized that they wanted to do a better job. One veterinarian with 38 years of working experience mentioned that *“we as vets were actually trained to euthanize. And if you have a diagnosis, yes, with a poor prognosis or something, then it should be euthanized. And today I look at it in a much more differentiated way, also because some people have come up to me and said that they would actually like to accompany the animal and not euthanize it.”* (Interview 8, Pos. 33) This is further supported by the following statement that highlights the aspiration to provide optimal care even though the animal cannot be cured anymore: *“[W]hy did I start as a vet? To be able to do more for the animals. Then I stand there and cannot do anything when they are dying or having to euthanize them myself. An incredible pain in me drove me to read other books, to think outside the box, to network with others, to see other points of view, to see indigenous points of view.”* (Interview 4, Pos. 191).

#### 3.1.3 Time pressure and “apparatus” medicine in today’s small animal practice

In addition to the discussion about euthanasia, some veterinarians also expressed concerns about the increasing time pressure and “apparatus medicine,” which not only leads to a potentially problematic overuse of diagnostic tests and overtreatment, but also neglects the aspect of appropriate care for animals with their disease. Some veterinarians draw parallels to the field of human medicine where the medicine is compared with “machinery” as stated by one veterinarian as follows: *“So I often hear that from people who do not want to get into such a […] machinery.”* (Interview 8, Pos. 159) Many interviewees expressed that *“[n]ot everything that is possible is also reasonable.”* (Interview 6, Pos. 23) and wished that veterinary medicine would step *“[…] away from this, so-called apparatus medicine towards a more humane medicine.”* (Interview 8, Pos. 155) This aspect is further supported by the following statement that underlines the necessity of palliative care and hospice service in today’s highly developed veterinary medicine: *“And I believe that end-of-life care takes on something that is less possible in, let us say, technologized medicine. Also, because it takes place in animal hospitals, where there is not the time, where the contact is perhaps less personal then when I’ve been looking after people for a long time.”* (Interview 8, Pos. 35).

In light of recent developments paired with the increasing time pressure on clinics and practices to treat the individual patient, some veterinarians reported that these developments not only motivated them to enter the field of hospice and palliative care, but also to look for alternatives to care for animals, e.g., homeopathy. For instance, one veterinarian who focused on homeopathy stated that it *“[…] then became more and more difficult for me and I also wanted to have more time for the client and not as it is in a small animal practice, where you have a quarter of an hour, 20 min, then the next one comes. And at some point, that was about eight years ago, I switched completely to homeopathy.”* (Interview 8, Pos. 17).

#### 3.1.4 The life experience of veterinarians and the associated change in perception of dying and death

Another aspect that was identified is a veterinarian’s life experiences. These experiences particularly triggered their endeavor to reflect on how patients should be treated in the last stages of their lives and how we should let animals die. For example, one veterinarian reflected on euthanasia decisions and explained that these considerations *“have become increasingly important with age.”* (Interview 14, Pos. 28) In particular, veterinarians with 25 years or more of experience, increasingly thought about “conventional” medicine and recognized their interest and motivation to work in the field of hospice and palliative care. One veterinarian with 28 years of professional experience stated *“for some time now, I’ve actually been wanting to get rid of conventional work more and more*, i.e.*, vaccinations and all that kind of work, and concentrate solely on end-of-life care. That’s what I’ve been doing for about four or five years now, it’s coincided with the […] middle of my life, to crystallize what my strength and my fire is. My inner fire.”* (Interview 9, Pos. 19).

### 3.2 Relationships

Veterinarians extensively talked about various aspects that shape not only relationships between the animal, the caregiver, and the veterinarian, but are also required to successfully care for a patient in this specific stage of life.

#### 3.2.1 Close emotional bonds between the caregiver and the animal and their effects on veterinary care

Most interviewees mentioned that caregivers who make use of hospice and palliative care usually have a close emotional bond to their animal. These animals are often described as a “family member,” “partner,” or even “child replacement.” Even though emotional closeness is of significance, at the same time many veterinarians stated that it is important that caregivers do not humanize their animal. For instance, one veterinarian argued: *“That’s an important point for me, on the one hand not to humanize them, but on the other hand to see them as a fully-fledged member [of the family].”* (Interview 20, Pos. 247).

In addition, some interview partners also highlighted that a certain level of emotional closeness is required to be a good “caregiver” and “attachment figure” for the chronically and/or terminally ill animal. Even though emotional attachment is important to be a good caregiver, energy, resources, time, and money are of significance, and if these are lacking, they may compromise animal welfare or caregiver’s wellbeing. Hence, these caregivers need to be able to handle the additional care required and be willing to compromise. If these things are not present or cannot be handled by the caregiver, a positive foundation for the required caregiving efforts is lacking. As one veterinarian described: *“And that tells me everything about people and everything […] about their relationship with their pets, right? Yes, and then there are just the people who cannot deal with pain, loss, and illness, right? Even with, oh God, I have to give medication every day, I have to do something […] I have to look after my pet. […] I cannot go away all the time at the weekend because he needs his tablets.”* (Interview 1, Pos. 87).

In addition, some veterinarians addressed the fact that a strong identification with the client’s “carer role” can have a major impact not only on the animal patient, but also on the caregiver. On the one hand, it can have the negative effect that they do not want to part with their pet and thus encourage overtreatment. On the other hand, it can lead to an enormous burden for the carer, as one veterinarian described as follows: *“I’ve also accompanied people where I was really at the point ‘Are you sure that you still want to and can accompany the animal like this?’ And wouldn’t it make sense to put him to sleep now, for your own sake? Because the animal wasn’t in a lot of pain, but it could not keep clean, kept losing stools, had to be carried out several times, even at night, and if you do that for a month or two, then everyone reaches their limits at some point.”* (Interview 20, Pos. 113).

#### 3.2.2 Empathy and trust guide relationships between the veterinarian and the caregiver

With respect to the relationship between the veterinarian and the caregiver, all veterinarians agreed that this specific service needs a certain level of empathy to guide as well as accompany the client while caring for their animal. Veterinarians practicing homeopathy additionally mentioned that the “chemistry” between them must be right in order to successfully provide this service. One veterinarian stated *“[…] first and foremost that the chemistry is right. That’s why I always say that from the beginning they have to get to know me, whether I suit them and the animal. And the animal also has to react to me in some way, because if an animal is afraid of me or panics […], then of course it’s difficult. So that’s important to me. Trust, that they simply have a good feeling, that they realize that I know what I’m talking about and that I’m available for them.”* (Interview 12, Pos. 55).

The aspect of trust was revealed as an important aspect by many veterinarians which in some situations can lead to intimate and personal relationships. For instance, a specialized veterinarian reports that *“And of course you somehow develop a more intimate relationship with the caregiver and it’s also quite a lot, so that people also tell you a lot of private things about their lives. [S]ometimes it’s a bit you have to digest, because sometimes it’s the difficult things that they tell you about their private lives – like the death of a husband or children, or cancer or something like that.”* (Interview 2, Pos. 73).

However, even though relationships can become familiar and personal, some veterinarians highlighted that it is important to keep a professional distance. One veterinarian explicitly stated that this professional distance must be maintained for one’s own salvation and that the consideration of relational aspects of the client is a service for which every veterinarian must demand money: *“So I keep my distance for my own well-being as best I can. But I try not to be cold in any way, […] I just stay correct. I’m not a first-name terms with the owner. But I also try to do it for the reason that you also have to think that it’s still a fee note that’s put down for services and for private conversations, five hours on the phone because you cannot decide whether to put a dog to sleep, what do you write for a fee note?”* (Interview 1, Pos. 137).

### 3.3 The aspect of communication

Veterinarians of this interview study provided insights into how they communicate with their clients and identified topics important to their clients.

#### 3.3.1 Channels of communication

Although one veterinarian who solely practices homeopathy reported that she often cares for clients abroad and therefore only needs a mobile phone to offer end-of-life care and hospice service, most veterinarians stated that it is important to personally communicate with the client during consultation hours or home visits on a regular basis. All veterinarians further reported that they used additional modes of communication including telephone calls or e-mails. In addition, some veterinarians were also in contact with their clients via mobile phone texting, videos and photos (messenger apps like WhatsApp). Some of the interviewees admitted that they offer far more opportunities for communication in this particular area of work.

#### 3.3.2 Topics of communication

When asked what clients want to communicate with their vets about, the study participants mentioned various aspects relating to both the animal and the caregivers. Many veterinarians reported that clients particularly want to talk about the patient’s quality of life, the avoidance of suffering and the right time for euthanasia. For instance, one veterinarian reported the following: *“The right time is a very, very important issue. Nobody wants it too early, and nobody wants it too late. And there is a lot of uncertainty. People are afraid of that. They’re afraid of missing the right time. They are afraid of losing time if they decide too early. And they are afraid that their animal will suffer if they decide too late. That’s actually the most important issue.”* (Interview, 6, Pos. 77).

In this context, the majority of veterinarians emphasized the importance of communicating transparently with the client about these aspects and not making false promises about cures or possible treatments, as described in the following statement: *“Of course, you have to play with very open cards, because what I assume about people does not always have to be true, yes? In other words, I always explain what options we have for dealing with the problem, with the illness, with old age. I can also say, and I’m often asked about this, what would you do if it were your own animal, yes? And then I can reveal my own attitude to it.”* (Interview 6, Pos. 57) This perspective is supported by the following statement from a veterinarian, which states: *“[I] do not want to sell hopes and that’s also basically a point where the communication with the caregiver has to be very honest, yes, that you say there is this limit, there is this age limit, where we simply know that the examinations may become more frequent, the treatment protocols change more often, simply because the kidney or liver values do not fit. So, you have more often contact with the patient’s caregiver […].”* (Interview 5, Pos. 43).

Although veterinarians indicated that the main questions and issues addressed by the clients focus on the animal patient, participants reported that topics can become very personal in this working context. A specialized veterinarian stated that *“[o]f course, they also talk about philosophical topics, spiritual topics, they reveal a lot about themselves to me. I see them crying, being desperate. Yes, lots of questions, they have lots of questions. Yes, it becomes very personal.”* (Interview 8, Pos. 57) In relation to private topics, one interview partner reported that *“Oh God! [….] We know everything about our owner. We know who has problems with their husbands, who has what … so everything. So, they like to tell us, and you often have to slow them down, we know the psychological deficits, the neurological deficits, the health deficits.”* (Interview 13, Pos. 89).

### 3.4 The aspect of time

The study participants generally agreed that time was one of the most important factors in hospice and palliative care, as one veterinarian put it: *“So time is really almost the most important thing for me, that I take the time and that the caregiver also has this time.”* (Interview 11, Pos. 45) Herewith, time has been discussed on different levels.

#### 3.4.1 Taking time for the patient and the caregiver

Veterinarians reported that in comparison to other veterinary services, more time is offered to both the patient and the client. One veterinarian stated that *“[…] you need far more time. So that’s … whereas the treatment of the animal usually only takes a few minutes and the care, for example the preparation of the plan, the care of the client, that probably accounts for 90 percent.”* (Interview 10, Pos. 57) The fact that the emotional support and care of the client takes time can be further illustrated by the following statement: *“[…] I take a lot of time and respond to the feelings, to these emotions, to the despair, to the powerlessness.”* (Interview 9, Pos. 97).

Furthermore, the interviews show that it is not only important to have enough time and to take time for the patient and, above all, for the client, but that this also increases the work satisfaction. Some veterinarians emphasized that this aspect was also a reason for leaving a practice or clinic and becoming a self-employed veterinarian. They were able to take the necessary time for individual patients and caregivers without being restricted by superiors or by schedule of back-to-back appointments. For instance, one veterinarian explained: *“You cannot have the information you need in 10 or 15 min to be able to give really comprehensive advice. And after 10 or 15 min, people do not have the space to really give you the most important information. […] So that was certainly a point where I said that’s not possible for me. And I have … I now have the opportunity through palliative care, through home visits, really in a safe way, I consciously take at least an hour and that makes such a difference for me and for the people. So, I have to say, that makes medicine fun for me again.”* (Interview 15, Pos. 26).

#### 3.4.2 Having enough time to say good-bye

Another level on which the aspect of time was discussed by many study participants was euthanasia. The veterinarians emphasized that euthanasia requires patience, calmness and privacy. One veterinarian explained how he deals with this in his practice as follows: *“Well, in principle, when we know that an animal is going to be euthanized, we block out a lot of time so that we do not rush to put the animal away and get straight on with the vaccination. In other words, you have to make sure that a certain rest period or quiet room is created, then you also communicate in the practice that you are not laughing out loud in the corridor or any such stories. So, yes, you have to try to create an oasis of calm so that the caregivers aren’t stressed by secondary noises […].”* (Interview 18, Pos. 98).

Further, mobile veterinarians, in particular, acknowledge that they are able to euthanize the animal at the caregivers’ home and that in comparison to practices and clinics they are able to take the time needed. For instance, one mobile veterinarian stated the following: *“[S]o when you go to the clinic, you have half an hour at most and then the issue is over. But I’m on site from one hour to three hours. […] So time is a really important factor, because then they want to tell you where the dog came from and how they got it and then the children come and say goodbye. And these are all things that I cannot say, now quickly, quickly, quickly, and I do not want that either, because it has to be a dignified farewell for everyone involved […]”* (Interview 12, Pos. 79).

#### 3.4.3 Dying and death is not plannable

In relation to the aspect of time veterinarians discussed that dying needs time, is unpredictable and *“[b]eyond control”* (Interview 9, Pos. 165) One veterinarian explained this as follows: *“[The animal] takes the time it needs. And that’s what I always say to people, to caregivers, we give the animal the time it needs to make its way.”* (Interview 9, Pos. 153) However, some veterinarians also addressed that the non-plannability can be challenging for them: *“But that’s actually a problem, yes, definitely. Because you cannot schedule it. It’s just the way it is. It happens the way it happens. There’s nothing I can do to speed it up or shorten it or lengthen it. It is the way it is.”* (Interview 4, Pos. 146–147).

In situations where veterinarians know that a patient will die or has to be euthanized soon, the aspect of time plays an important role since many veterinarians want to be available. Although it can have an impact on their private life, some veterinarians stated that they are reachable via their private mobile phone and often around the clock. One veterinarian said: *“And it’s very much appreciated, I have to say, it’s very much appreciated by the client that you say I’ll come to your home for this point, or that you say I’m also available for you at the weekend, even if I’m not on duty. So that is something that is very, very much appreciated. […] That’s why I’m prepared to say that when we get to the home stretch, when the time comes, people can contact me and I’ll give them the phone number where I can be reached.”* (Interview 6, Pos. 15).

Even though time was considered as an important aspect, taking time for the patient and the client in this working field is often challenging to charge for. A mobile veterinarian said: “*And how am I supposed to account for that? It does not work at all. I invest a lot of time in it and of course I try to bill it properly, but I cannot bill it the way I often have to, can I?”* (Interview 12, Pos. 79) The challenge of charging enough was also emphasized by a self-employed veterinarian who provides house calls: *“It’s time-consuming. I spend a lot of time doing things that are not on the fee schedule. It’s something that you either say I do because I feel morally obliged to do it, but I do not get paid for it, in other words I do not get compensated in monetary terms. […] So, the process, the time you spend with people, yes, I have to say, it’s time-consuming and you have to decide whether you want to take it with you into the next life as karma or whether you want to be paid for it.”* (Interview 6, Pos. 91).

### 3.5 Infrastructural aspect

#### 3.5.1 Providing home visits

Turning to infrastructural aspects, a prominent topic was the provision of home visits. While three mobile veterinarians included in this study only provide home care, also all other veterinarians agreed that the provision of home visits is very helpful in specific situations. For example, veterinarians reported that it is helpful to know where the animal lives in order to give the caregiver better advice. For example, in order to discuss the living environment or where ramps could be placed for the animal to make walking easier and less painful, as explained by one veterinarian as follows: *“And I can also see how the animal moves in the environment, so I can perhaps see more easily, aha, that’s where we have the problem, we might have to solve it differently. […] And accordingly, you can individualize the support much better, because depending on the problem, here we are back to the same topic with the individual care plan, depending on the circumstances, I can handle something much better with an animal in a garden apartment than now on the 5th floor in the middle of the city.”* (Interview 16, Pos. 116).

Further, veterinarians who offer homeopathy emphasize the advantages of home visits: *“So one of the advantages for homoeopathy is of course that I can see the animal in the environment where it lives, that I can take a better look at how it is doing there, observe how it behaves at home in its familiar surroundings, which is certainly a great advantage, especially for homeopathy. I can also experience the caregiver in his natural environment.”* (Interview 20, Pos. 189).

Euthanasia was frequently discussed by veterinarians in relation to home visits. Veterinarians revealed two main reasons for this. First, transport of the patient can be avoided, which reduces stress for the animal and the caregiver during the last hours of life. Second, euthanasia at home can be much more pleasant for the caregiver, as the situation is more intimate, calmer than in a practice, and further, the owners can give free rein to their emotions more easily if necessary. For instance, one veterinarian stated the following in relation to both aspects: *“The advantages are quite clear – for the dog, a familiar environment, a relaxed atmosphere, not the uproar that the animal already has at the vet. And the same for the humans, right? They do not need to feel like they are being watched, they can pursue their emotions, whereas they tend to be inhibited in the practice.”* (Interview 6, Pos. 125).

#### 3.5.2 Providing a “safe space” in the practice

In addition to the discussions surrounding home visits, many veterinarians would like to see a better infrastructure in their practice to better care for palliative patients. They would like to provide a “safe space” for the animal and caregiver, especially if the patient has to be euthanized in the practice: *“So I would like to have a room for these farewells. […] So that people really have a retreat area where they are completely undisturbed.”* (Interview 5, Pos. 101) One vet would even like to set up and run an institutionalized hospice for animal patients: *“[A]nyway, what would be nice would be a hospice for animals, let us put it that way. Just like it is in human medicine, it would be cool to simply say, okay, they can come there and be shown how to do it, or they can die there, […] or for animals who do not have caregivers, but are alone on this planet […]”* (Interview 12, Pos. 107) Even though most vets would like to see a better infrastructure in terms of premises, one veterinarian reported: *“It does not need infrastructure, it needs the phone, or, it needs the heart, it just needs me as a person.”* (Interview 9, Pos. 179).

#### 3.5.3 Flexibility through self-employment

Even though some veterinarians stated that they would like to see more of their colleagues offering hospice and palliative care for patients and their caregivers, the majority of veterinarians stated that they appreciate working alone as it brings a certain freedom and flexibility to their working day. However, veterinarians who work with veterinary assistants acknowledge, in particular, their support in client communication, as one veterinarian emphasized: *“[Veterinary assistants] are with us, they are everywhere, they help a lot, they are especially involved in client communication, that is very important […]”* (Interview 19, Pos. 19) In addition to that, some interviewed veterinarians see the high potential of a veterinary assistant’s service in providing additional support for caregivers at home who care for palliative patients.

## 4 Discussion

Our current understanding of the different demands and needs of working in the field of small animal hospice and palliative care is largely anecdotal. This interview study identified that, in particular, personal experiences with their terminally ill pet or negative experiences either during training or at the beginning of their professional life motivated veterinarians to provide and/or specialize in hospice and palliative care. Further, empathetic-driven relationships appear to be of importance, however, veterinarians highlighted that keeping an emotional distance to the patient and caregiver is significant to provide successful care. Veterinarians expressed that having time for their patients and the patients’ caregivers was one of the most important aspects of working in this field.

Participants of this study reported that previous negative experiences with their own animal(s) and negative experiences during training or at the beginning of their professional life related to topics like euthanasia, triggered their interest and motivation to build competences in the field of animal hospice and palliative care. Further, we identified that life experiences and associated changes in the perception of dying and death can stimulate, in particular, older veterinarians to reflect on how they would like to care for their patients in the last stages of life. In the existing literature, research that focuses on the investigation of private experiences affecting professionals’ practice can be mainly found in fields related to therapeutic work (in the field of human medical care) (30–32) or pedagogy (33–36). For instance, based on qualitative research findings, Jensen (30) identified that besides educational training and practical experiences, personal and private life forms an integral part of building competences and interests in therapists’ work life. That personal and private experiences are often neglected in discussions of clinical competence has been emphasized repeatedly by various researchers in recent years (30–32). Consequently, private experiences should not ‘silently’ inform practice, but rather actively foreground ‘an epistemology of personal knowledge’ in a professionals’ life (32). One possible explanation for the more advanced engagement with personal and private life as an integral part of therapists’ professional skill-building could be that, unlike veterinarians, therapists (for humans) benefit at least in some countries from professional supervision, which allows for some debriefing and consideration of personal aspects that can support their work. Even though studies in the field of veterinary medicine have shown that personal experiences with own animals are an important source of motivation to study veterinary medicine or affect students’ choices among different career paths (37), currently, little is known on how professionals’ private and personal experiences, e.g., with their own animals, shape their clinical practice during their career.

In addition, not only private experiences, but also educational experiences motivated veterinarians to work in the field of animal hospice and palliative care. In relation to this, handling of end-of-life decisions including euthanasia were critically discussed by some interviewees by pinpointing the fact that euthanasia was considered as the option in case of a poor prognosis without considering possible alternative treatments including palliative care. While some decades ago, euthanasia was often the only option available to avoid suffering of a chronically and/or terminally ill animal, (9), discussions and decisions regarding the assistance of death in animals have undoubtedly changed. The reasons for these changes are manifold and range from the general perception and integration of pets into today’s society (38), to the increasingly emotional relationships between clients and animals (39, 40), to changing legal regulations on the killing of animals [e.g., in Austria and Germany a “justified reason” is needed to kill an animal (41)], and the constant further development of veterinary medicine and the associated improvement in patient care (2–4). The changes in relation to these various aspects not only shift the question of when euthanasia should be performed but can be seen to provide fertile ground for both—the increasing personal and professional aspirations of veterinarians to care for patients suffering from terminal illness, and hence, the associated development of the field of animal hospice and palliative care in small animal practice today.

Although developments in small animal practice have led to the improvement of patient care, the results of this study show that veterinarians are concerned about these advancements to a certain extent. In relation to this, parallels can be drawn to debates in human medicine, where enormous developments in and the implementation of, e.g., intensive critical care units ignited a heated debate on how far medicine should go in patient care (42). In recent years, these debates have found their way into veterinary medicine (2, 3, 43, 44). For instance, in a study among veterinary anesthetists, most respondents were extremely (17%) or moderately (43%) concerned about intensive care units, and over 70% of the anesthetists agreed that a wider debate is needed to address ethical issues in this field of care (44). The demarcation from these developments, which has been discussed as “apparatus veterinary medicine” by our interviewed veterinarians, was identified as a further source of motivation for our interviewed veterinarians to offer hospice and palliative care service. “Apparatus veterinary medicine” not only stands for an excessive use of technology with a tendency toward futile interventions, it also includes criticism of a system in which the veterinarian has too little time for the animal and the caregiver. Palliative care stands at the other end of the spectrum of medicine and in contrast to the technologized medicine used in the intensive care units. In a letter to the editor entitled “Palliative Medicine and Intensive Care Medicine—Two Sides of the Same Coin?,” the physicians Clemens and Klaschik (42) write: “Superficially, palliative medicine and intensive care medicine seem to be at the opposite ends of care; one is known as ‘talking medicine’ and the other as ‘apparatus medicine’.” This contrast becomes evident based on our data indicating that veterinarians highlight the importance of good communication including having enough time to talk with the caregiver about various aspects surrounding the animal with a life-limiting disease. Even though hospice and palliative care is discussed as a counterpart of the increasing advancements of patient care including the use of technical equipment, it is important to note that both ends of the spectrum of patient care share some commonalities. In relation to this, Clemens and Klaschik (42) state that, in human medicine, both disciplines aim to “stabilize patients to facilitate discharge to their homes” and to work in interdisciplinary teams that include specialists who can handle emotional and physical strains. Further, they highlight that both disciplines require not only the ability to reflect on end-of-life issues, but also to discuss and choose realistic goals of care and to communicate openly with patients and relatives (42).

The participants of our interview study confirmed the importance of open and transparent communication in the field of hospice and palliative care. That good communication strategies are needed to provide optimal care for both the animal and the caregiver, and that in turn can have a positive impact on caregivers’ adherence can be confirmed by various research (12, 22, 45–49). For instance, Moses (12) highlights that it is important to have a clear communication strategy that avoids euphemisms and clinical jargon, is empathetical and non-judgmental, and gives special attention to the feelings of the caregiver. This is further underpinned by Brown and colleagues (47) who state that relationship-centered communication including careful listening and allowing clients to speak about their concerns and feelings is important in building trust and developing an appropriate spectrum of care. Our findings add to this conclusion. They indicate that empathy and trust guide relationships between the veterinarian and the caregiver and is considered as a relevant aspect to successfully care for both the animal and the caregiver. In addition to that, research shows that good communication can have a vast impact on owner compliance. For instance, Lue and colleagues (49) found that 70% of surveyed pet owners who believed that their veterinarian communicates well followed the veterinarians’ recommendation. On the contrary, when pet owners perceive communication to be worse, only 5 in 10 pet owners continued following recommendations (49). Furthermore, their data show that the difference in compliance was even greater between clients who did not believe their veterinarians were trying to sell them unnecessary products or services versus those who did (49). Since caregiver compliance is a particularly important aspect in the field of palliative care, where a great deal is demanded of the caregiver, our results underline the importance of a good communication strategy in this area of work.

A further aim of this study was to identify what caregivers want to talk about with their veterinarian. Our findings show that besides patient-related questions that address quality of life, avoidance of suffering or the right time to euthanize the animal, participants reported that conversations can turn very personal. Herewith, veterinarians stated that clients often reveal private issues or want to talk about fundamental topics that go beyond the medical aspects. In relation to this, the authors of the comprehensive book entitled “Hospice and Palliative Care for Companion Animals—Principle and Practice” state among other things that “[a]nimal hospice also addresses mental health—the psychological, emotional, social, and spiritual needs of the human caregivers in preparation for the death of the animal, and subsequent grief” or that palliative care “also provides caregivers with emotional and spiritual support and guidance.” (50) Against the background of these observations, our results confirm that veterinarians not only experience these specific needs of the caregivers, but also recognize that this is an important part of their work in the field of hospice and palliative care.

While previous research has emphasized the importance of transparent and empathetic communication in relation to various issues raised by caregivers (12, 22, 45–49), the question of which communication channels veterinarians use has, to the authors’ knowledge, not been empirically investigated to date. It was expected that channels of communication may change when caring for animal at the end of their lives. This can be confirmed based on our findings. Almost all interviewed veterinarians highlighted the importance of personal communication in a face-to-face situation with the clients on a regular basis. However, our analysis also show that veterinarians provide more possibilities to communicate and get in touch with the caregivers when compared to traditional care of animals. Besides the personal consultation during consultation hours or home visits, phone calls, writing e-mails or short messages as well as sending photos and pictures via mobile phones are offered by the majority of veterinarians. Offering these ways of communication are a form of ongoing support that are not just messages, but provide continuity of support for clients caring for their ill pet, which requires work and time on the part of the veterinarian.

There is no doubt that these additional channels support clients in the care of their pet, may promote clients’ adherence and make it easier for veterinarians to stay in contact with caregivers, especially during critical phases. However, this offer can also have its challenges since the additional provision of channels to get in touch with the veterinarian can be very demanding. In relation to this, our data reveal that the unpredictability of dying and death requires accessibility of veterinarians—sometimes even around the clock. This is time-consuming and even affects the veterinarian’s private life. Some veterinarians stated that they are reachable via their private phone number when they know that the patient will die or has to be euthanized soon. Keeping this in mind, veterinarians can feel torn by their aspiration to provide the best care for the animal and the caregiver on the one hand, on the other hand, to take care of their private and personal needs. This challenging aspect has been further identified by Hoffmann and Dickinson (15) who found that veterinarians working in the field of hospice and palliative care mentioned “the mental aspect,” “personal burn out,” “self-care,” “isolating,” or “dealing with my sorrows” as really demanding aspects during the provision of end-of-life care. At this juncture, veterinarians should acknowledge the importance of maintaining self-care including managing time commitments of care and balancing caregiving with ongoing responsibilities as addressed by Cox and Goldberg (51). They suggest that veterinarians working in the field of animal hospice and palliative care should consider steps to manage their time including making lists of tasks that need to be accomplished, balancing the effort, taking breaks or keeping track of progress by crossing things off the list as they are completed (51). In addition, they highlight that besides using scheduling programs and tools to manage time more effectively, transparent talks with the boss or colleagues on time management should be considered, as well as asking for help to balance ongoing responsibilities (51). While checklists and tools can offer some structure to veterinarians’ work life, they cannot fully address the issue of burnout, especially considering the constant on-call nature of their work. This constant availability arises from their deep commitment to providing compassionate end-of-life care for animals, a commitment aligned with their personal values. Recognizing the unique challenges of this profession, it’s crucial that veterinarians actively seek out and implement strategies and structures that promote sustainable and healthy work practices.

Interestingly, the need for more staff in the practice to better provide animal hospice and palliative care was rarely discussed by our study participants. Even though some of our interviewed veterinarians would like to have more colleagues who provide animal hospice and palliative care including the option of a veterinary nurse service that additionally provide support for caregivers caring for terminally ill animals, our findings show that self-employment helps them to be more flexible in time management and patient care. Although self-employment increases flexibility in time and patient management, it is to be expected that working alone will result in no or fewer opportunities to discuss exhaustive periods. This in turn can increase the risk of overlooking signs of fatigue and overwork. With this in mind, it is important that veterinarians consider aspects of self-care management by reflecting on their own time and energy resources while caring for animals and their caregivers.

It was expected that in the interview study, the provision of hospice and palliative care would be closely tied to the provision of home visits. This expectation can be confirmed by our results: regardless of whether the veterinarians work as mobile veterinarians only, the provision of house calls was a prominent topic in most of the interviews. Herewith, veterinarians referred to positive aspects from the perspective of the animal and the caregiver such as that home visits help vets to give better advice or that the animal does not have to be transported to the practice. Further, home visits reduce fear, anxiety and distress in patients but also increase accessibility of veterinary care to animal caregivers who may have mobility and/or transport difficulties. In addition, some veterinarians further reported that it is more intimate, calmer at home, and that caregivers can give free rein to their emotions especially in cases where the animal has to be euthanized. There is no doubt that at-home care allows the animal and the caregiver a higher level of privacy and comfort (52) when compared to consultations in the practice rooms. This experienced difference is further supported by the finding that some veterinarians wish to provide a “safe space” in their practice in order to have an extra room where caregivers can say goodbye to their animal peacefully and privately.

Turning to the veterinarians’ discussions about the aspect of time, our data suggests that having enough time and taking time for the care of the patient and the caregiver is one of the most important and valuable aspect in the field of animal hospice and palliative care. This can be supported by findings of Hoffmann and Dickinson (15) who identified that not having enough time was one of the most challenging aspects for veterinarians. In addition, our findings reveal that the high value of time is strongly related to three aspects: first, veterinarians’ motivation to work in this field, second, to the development of relationships and third, to the provision of home calls. For instance, we found that the increasing time pressure in veterinary practice has led some veterinarians to work independently and specialize in the field of hospice and palliative care. The fact that in modern veterinary medicine little time is available for diagnosis and treatment is also shown by a study by Belshaw and colleagues (53). Their findings show that both owners and veterinarians are concerned about rushing and minimizing discussions to keep consultations on time. That in turn does not only impact the quality of care, but also negatively affects the owner-veterinarian relationship (53).

With respect to relationships, we identified that time is an essential factor to provide patient-centered care including emotional support for the owner. This is also reflected in the great need for veterinarians to give caregivers enough time when they say goodbye to their animals. Our interviewed veterinarians working in mobile practice highlighted that their choice to care for the animals and their caregivers at home meets this requirement and their aspiration of having and taking enough time. Even though time was valued by all veterinarians, we identified one specific challenge in relation to this: regardless of whether veterinarians work as mobile veterinarians or not, they reported the challenge to charge for this time. In particular, the challenge to charge the caregiver refers to time veterinarians spend on non-medical aspects, for instance, providing the caregiver enough time for conversation after euthanasia of the animal. It can be assumed that the problems with billing these conversations are partly due to mental barriers on the part of the veterinarians. It could be that they sometimes have a bad conscience when billing these services or believe that the owner may misinterpret this as a kind of “friendly service.” Such problems of billing can have a negative impact on the veterinarian’s income. This can be partially supported by the findings of Hoffmann and Dickinson (15), who found that while the financial rewards of end-of-life veterinary care were not emphasized by veterinarians in their study, self-employed veterinarians struggled with financial problems in this area. Whereas the issue of cost of care including medications, diagnostics, environmental modifications has been addressed (51), as well as possibilities that help owners to cover costs, e.g., uptake of pet health insurance, we believe that more discussions and support are needed for veterinarians when it comes to the challenge of charging time. As our result suggests that time is one of the most important aspects, we encourage veterinarians to not hesitantly, but rather self-confidently charge money for their provision of time and to discuss this issue transparently with caregivers from the beginning. In addition, it would be important that, for example, fee regulations, to which veterinarians practicing in Germany must adhere, also include charging for consultations on an hourly basis or per unit of time in order to overcome such barriers.

While veterinary medicine has advanced significantly in terms of medical treatment, structural changes and economic pressures have inadvertently side-lined crucial aspects of care. This includes dedicating sufficient time to both the patient and the owner, addressing the unique bond between humans and animals, and prioritizing quality of life over solely focusing on cure. Given the ongoing advancements in veterinary medicine, the field of palliative care and hospice is poised for increased importance. To meet this growing need, we require veterinarians who not only establish the framework for this type of care and explicitly address it in their communication (for example, on the practice website), but also actively engage caregivers in its use and implementation. Herewith, time and relationships are key to creating an ideal environment for “talking medicine,” a crucial component of successful end-of-life care.

Although it is a strength of the present study that participants were carefully selected thorough search of the German, Austrian, and Swiss populations of small animal veterinarians, the study also has limitations. First, to employ a comparable sampling strategy in the three countries, we made use of online classified directories in order to find veterinarians who specialized in the field of animal hospice and palliative care and/or explicitly mentioned and advertised this service on their practice website. Even though online classified directories provide a good overview of practicing veterinarians and practices, those veterinarians who are not using practice website or are not displayed in the used directories has not been covered by our sampling strategy. Hence, a coverage error exists between the veterinarians displayed online and the ‘true’ number of small animal veterinarians and can lead to a possible bias. Further, it can be expected that even though veterinarians did not explicitly mention this service on their website that they provided hospice and palliative care to a certain extent; especially as the boundaries here are not entirely clear. This can further lead to exclusion of veterinarians who did not advertise the service, but still offer it. Second, great care was taken to ensure that different aspects such as type of practice, employment status, gender and type of specialization were considered. However, it was difficult to recruit male, salaried and mobile veterinarians, which may lead to some bias in our results. Third, it is important to note that some of the recruited interview partners considered themselves as “spiritual” which actually drove their change from traditional small animal practice into animal hospice and palliative care. Although this need not be considered a limitation *per se*, it may affect their attitudes toward the aspects analyzed in this study. Fourth, interviewers may steer interviews differently, knowingly, or unknowingly, in a direction which biased responses, or avoided unwanted outcomes. Further, the different professional backgrounds of both researchers (S.S. veterinary background, C.D. philosophy background) may have impacted interviews in which the medical knowledge of one researcher brought a different understanding of certain terms used during the interviews. In the present study, we attempted to minimize bias by developing the interview guide with both researchers (S.S. and C.D.) and spent time and effort discussing the interview process. In addition, two pilot interviews were conducted (one by S.S. and one by C.D.), which were subsequently discussed and analyzed. The fact that the study was carried out in German-speaking countries including Germany, Austria, and Switzerland may also mean that some results will be less relevant in other countries. This may be because of cross-country differences in the structural set-up of small animal practice and of animal hospice and palliative services, such as the comparatively high number of self-employed veterinarians in the three countries studied.

This interview study as a qualitative method was chosen to shed light on veterinarians’ motivation to build competences in the field of animal hospice and palliative care and to empirically investigate various aspects such as relationships, communication, time, and infrastructure that have been considered of particular relevance for the field but were not researched in past studies. This interview study including 20 individual focused interviews is explorative in nature without making the claim of generalizability or representativity of results. Therefore, we recommend that a quantitative study, e.g., questionnaire-based survey, should be undertaken to provide representative results relating to the themes presented here. In addition, further research on this subject could focus on cross-country disparities to compare the effect of different legal backgrounds, different organizational structures, and different professional norms. Based on our study, we further suggest that future research should make an in-depth investigation of the compatibility of the private and professional lives of veterinarians working in hospice and palliative care and possible emerging challenges and conflicts.

## 5 Conclusion

The results of the interview study show that veterinarians’ experiences in particular, including personal experiences with their own pets or during their training or professional life, motivated them to provide hospice and palliative care. Keeping this in mind, our results suggest that more research is needed to further investigate to what extent professionals’ previous or even current private experiences may impact and shape their professional career and builds an important source of motivation to leave certain career paths or focus on specific fields of veterinary medicine.

Although empathy-driven relationships between caregiver and veterinarian appear to be important, our results show that emotional distance from both patient and caregiver is important for successful care. In addition, the increasing frequency and opportunities offered for communication with caregiver requires a high time commitment on the part of the veterinarian. Having time for the patient and their family is therefore one of the most important and valued aspects of working in this field. However, it is important that veterinarians consider aspects of self-care management by thinking about their own time and energy resources while caring for animals and their caregivers.

## Data availability statement

The original contributions presented in the study are included in the article/Supplementary material, further inquiries can be directed to the corresponding author.

## Ethics statement

The studies involving humans were approved by the Ethics Committee of the Medical University of Vienna. The studies were conducted in accordance with the local legislation and institutional requirements. The participants provided their written informed consent to participate in this study. Written informed consent was obtained from the individual(s) for the publication of any potentially identifiable images or data included in this article.

## Author contributions

SS: Conceptualization, Data curation, Formal analysis, Funding acquisition, Investigation, Methodology, Project administration, Resources, Software, Visualization, Writing – original draft. SA: Investigation, Writing – review & editing. CD: Conceptualization, Data curation, Investigation, Methodology, Software, Validation, Writing – review & editing.

## Footnotes

^1^ 11880.com

^2^ local.ch

^3^ herold.at
