# Supplementary material for: “Veterinary medicine is not finished when I have diagnosed an incurable disease, that’s when it starts for me.” A qualitative interview study with small animal veterinarians on hospice and palliative care
Source: Front Vet Sci. 2024 Sep 5;11:1440404. doi: 10.3389/fvets.2024.1440404 (PMC11410772; doi:10.3389/fvets.2024.1440404)
Supplement: Supplementary file 2 [file Table_2.DOCX]

Supplementary Material 2

# Detailed description of parts and themes of the interview guide relevant for the present study

First, each interview began with an opening question in which veterinarians were asked to talk about their professional backgrounds and careers. After this opening question, the first theme in the first part of the interview guide aimed to elicit reasons to specialize in hospice and palliative care and/or offer this explicitly. In addition, the first theme sought to reveal insights into what it entails for veterinarians to provide palliative care for an animal and/or to accompany the patient during its death. Further, participants were asked about the role of hospice and palliative care in today’s small animal practice which is shaped by enormous developments with respect to technology and methods to improve patient care, and what they think it takes to be a ‘good’ veterinarian in the field of hospice and palliative care. The second theme of the first part of the interview guide focused on relationship constructions and characterizations (caregiver-animal; caregiver-veterinarian, veterinarian-animal) in hospice and palliative care. In addition, participants were asked to take a stand towards the following pointed statement: “Hospice and palliative care for animals is nothing more than a service for the pet owner. The animal gets nothing out of it.”

The second part was structured around communication and times aspects in this field. The overall aim was not only to identify the role of communication and time, but also changes with respect to these two aspects. In addition, interviewees were asked whether it makes a difference to them to lose a patient by natural death or by euthanasia. During the third part, veterinarians were questioned about infrastructural requirements including monetary and structural aspects; as well as changes to caring for the animal in its home environment.
