# Supplementary material for: “Veterinary medicine is not finished when I have diagnosed an incurable disease, that’s when it starts for me.” A qualitative interview study with small animal veterinarians on hospice and palliative care
Source: Front Vet Sci. 2024 Sep 5;11:1440404. doi: 10.3389/fvets.2024.1440404 (PMC11410772; doi:10.3389/fvets.2024.1440404)
Supplement: Supplementary file 3 [file Table_3.DOCX]

Supplementary Material 3

# Final Code List

| **CATEGORY 1:**  **H**OSPICE AND **P**ALLIATIVE CARE | **HP** | **Category 1 focusses on several aspects related to hospice and palliative care including a) veterinarians’ motives, b) significance of this field and demarcation to other fields, c) occurring challenges, d) death and euthanasia, and e) alternative medicine.** |
| --- | --- | --- |
| HP: motives | HP-MOT-EXP | Identified reasons and key experiences why veterinarians   1. became a veterinarian and interested in veterinary medicine including their aspirations 2. have specialized/ or are specializing in the field of hospice and/or palliative care and/or 3. explicitly offer hospice and/or palliative care on their website. |
| HP: demarcation to “classical curative fields” and/or other colleagues | HP-DEMARC | Identified   1. indices of demarcation to “classical curative fields” and/or 2. other colleagues. |
| HP: challenges | HP-CHAL | Identified challenges for veterinarians due to their work in the field of hospice and palliative care. |
| HP: natural death | HP-NAT | Identified   1. ways of thinking and feelings about natural death in small animal practice 2. and challenges related to the natural death of animals. |
| HP: euthanasia | HP-EUT | Identified   1. ways of thinking and feelings about euthanasia in small animal practice 2. decision-making processes and related challenges in regard to euthanasia. |
| HP: decision-making and challenges | HP-DEC | Identified components relevant for decision-making in general (including relevant stakeholders involved) and possible occurring challenges |
| HP: alternative medicine and complementary medical approaches | HP-ALT | Identified ways of thinking about and the use of alternative medicine (e.g. homeopathy, TCM) and complementary medical approaches (e.g. physiotherapy) in the field of hospice and palliative care and its positive and negative effects in the field of hospice and palliative care. |
| HP: other aspects | HP-OTHER | Other aspects of hospice and palliative care, which give an indication of what veterinarians mean or think of when they talk about hospice and palliative care. |
| **CATEGORY 2:**  **AN**IMAL-BASED FACTORS | **AN** | **Category 2 refers to aspects related to the animal patient.** |
| AN: capacities and effects | AN-CAP | Identified   1. characteristics and attributes of the animal patient (e.g. species, age, character of the animal) 2. indices of impact of animal patient’s attributes and characteristics on decision-making processes. |
| AN: health status | AN-HEALTH | Identified   1. statements focussing on animal patient’s health status (e.g. medical information) 2. indices of impact of animal patient’s health status on decision-making processes. |
| AN: guiding principles | AN-PRIN | Identified statements highlighting guiding principles (e.g. quality of life, avoidance of suffering, protection of life, welfare of the animal) and its significance in decision-making processes. |
| **CATEGORY 3:**  **VET**ERINARIAN-BASED FACTORS | VET | **Category 3 refers to aspects related to the veterinarian.** |
| VET: biography and career | VET-BIO | Identified aspects related to the veterinarians’ biography that has an impact on their career development and professional life. |
| VET: role of having pets | VET-PETS | Identified aspects related to their role and experiences made as animal owner on their professional life. |
| VET: skills and competencies | VET-SKILLS | Identified skills and competencies   1. to (successfully) work in the field of hospice and palliative care and 2. that go beyond the “classical” skills and competencies of veterinarians |
| VET: characteristics and (psychological) capacities | VET-CHAR | Identified   1. special features with respect to characteristics and psychological capacities of the veterinarian 2. indices of psychologically challenging experiences (e.g. burnout, mental/psychological stress) for the veterinarian |
| VET: Boundary work | VET-BOUND | Identified aspects and strategies with a view to demarcation between professional and private life. |
| VET: economic aspects and challenges | VET-ECO | Identified   1. economic aspects (e.g. the need to earn money) in regard to the veterinarian, 2. challenges with respect to profitability/economy of veterinary medicine (e.g. billing of services, GOT (GER: Gebührenverordnung) 3. veterinary medicine as service provision sector |
| **CATEGORY 4:**  **CL**IENT-BASED FACTORS | **CL** | **Category 4 refers to aspects related to the client.** |
| CL: characteristics and attributes | CL-CHAR | Identified   1. characteristics and attributes of the client (e.g. gender, age, character of the client) 2. indices of impact of client’s attributes and characteristics on decision-making processes. |
| CL: expectations and explanations | CL-EXPEC | Identified   1. expectations of clients including described expectations of clients towards veterinary services in hospice and palliative care, towards veterinarian in individual cases or possible inappropriate expectations of clients towards possibilities in hospice and palliative care 2. explanation for increasing/inappropriate expectations (e.g. clients compare with possibilities and progress in human medicine, further sources of information), status of the animal patient (family member) or living / private circumstances of clients)) |
| CL: emotional aspects and effects | CL-EMO | Identified   1. emotional aspects and emotionally driven situations by clients in hospice and palliative care 2. effects of client’s emotion during consultation and on decision-making processes |
| CL: financial aspects and effects | CL-FIN | Identified   1. financial aspects and financially-driven situations by clients in hospice and palliative care 2. effects of client’s financial background on decision-making processes |
| CL: disagreement and effects | CL-DIS | Identified   1. aspects related to disagreement among the client and the veterinarians 2. indices of impacts of disagreements among the client and the veterinarian on decision-making processes |
| **CATEGORY 5:**  **REL**ATIONSHIPS | **REL** | **Category 5 refers to relationships and relational aspects between the animal, the client and the veterinarian.** |
| REL: animal-client | REL-AC | Identified relational aspects and characteristics of relationships between the client and the animal patient. |
| REL: veterinarian-client | REL-VC | Identified relational aspects and characteristics of relationships between the veterinarian and the client. |
| REL: veterinarian-animal | REL-VA | Identified relational aspects and characteristics of relationships between the veterinarian and the animal. |
| REL: changes and effects | REL-CHAN | Identified  a) changes for practicing veterinarian in their relationships to their animal patient and the client due to their work in hospice and palliative care  b) indices of impact of changing relationships between the veterinarian, the client and the animal |
| REL: challenges | REL-CHAL | Identified emerging challenges for veterinarians in their relationships due to their work in hospice and palliative care and indices of impact of challenges on veterinarians. |
| REL: dealing | REL-DEAL | How veterinarians deal with challenges related to relationships (*conceptual* [veterinarians recognize fields of tension, but react rationally] versus *emotional* [veterinarians not only recognize the challenging situation but rather this field of tension stressed them out] |
| **CATEGORY 6:**  **COM**MUNICATION | **COM** | **Category 6 refers to aspects related to communication.** |
| COM: types of communication | COM-TYPE | Identified types of communication (interpersonal communication; communication via technical devices) with clients for consultation in hospice and palliative care. |
| COM: topics client-based | COM-TOP-CL | Identified topics and themes focussing on the client during consultations (e.g. discussion about private issues) |
| COM: topic animal-based | COM-TOP-AN | Identified topics and themes focussing on the animal during consultations. |
| COM: challenges | COM-CHAL | Identified emerging challenges related to communicational aspects in hospice and palliative and indices of impacts of challenges on veterinarians. |
| COM: dealing | COM-DEAL | How veterinarians deal with communicational challenges (*conceptual* [veterinarians recognize fields of tension, but react rationally] versus *emotional* [veterinarians not only recognize the challenging situation but rather this field of tension stressed them out] |
| **CATEGORY 7:**  Aspects of **TIME** | **TIME** | **Category 7 refers to aspects related to the aspect of time.** |
| TIME: role in consultation | TIME-ROL | Identified  a) components related to the aspect of time (e.g. duration, needed time, planning)  b) indices of impact of time during consultation and on decision-making processes |
| TIME: quality | TIME-QUAL | Identified components related to the quality of (lived and remaining) time of the animal patient. |
| TIME: quantity | TIME-QUAN | Identified components related to the quantity of time of the animal patient. |
| TIME: challenges | TIME-CHAL | Identified emerging challenges related to the aspect of time in hospice and palliative and indices of impacts of challenges on veterinarians. |
| **CATEGORY 8:**  **INF**RASTRUCTURE | **INF** | **Category 8 refers to aspects related to the infrastructure.** |
| INF: changes and implementation | INF-CHAN | Identified   1. occurring changes with respect to needed infrastructure in hospice and palliative care 2. implementation of hospice and palliative care |
| INF: house calls and positive effects | INF-HC-POS | Identified positive effects related to the provision of house calls in the field of hospice and palliative care |
| INF: house calls and challenges | INF-HC-CHAL | Identified challenges related to the provision of house calls in the field of hospice and palliative care |
| **CATEGORY 9:**  **DEATH** AND **SOC**IETY | **DEATH-SOC** | **Category 9 refers to components of dying, death and society.** |
| DEATH-SOC: (changing) perception | DEATH-SOC-PERC | How veterinarians perceive the issue of dying and death in society and possible changes in their perception due to their work as veterinarian. |
| DEATH-SOC: effects of dying companion animals | DEATH-SOC-EF | Identified effects of dying animals on societal perceptions of dying and death. |
